# Virtual or In-Person: A Mixed Methods Survey to Determine Exercise Programming Preferences during COVID-19

**DOI:** 10.3390/curroncol29100529

**Published:** 2022-09-20

**Authors:** Kirsten Suderman, Tara Skene, Christopher Sellar, Naomi Dolgoy, Edith Pituskin, Anil A. Joy, Susan Nicole Culos-Reed, Margaret L. McNeely

**Affiliations:** 1 Department of Physical Therapy, University of Alberta, Edmonton, AB T6G 2G4, Canada; 2 Department of Oncology, Faculty of Medicine, University of Alberta, Edmonton, AB T6G 2R3, Canada; 3 Cross Cancer Institute, Alberta Health Services, Edmonton, AB T6G 1Z2, Canada; 4 Faculty of Nursing, University of Alberta, Edmonton, AB T6C 1C9, Canada; 5 Cumming School of Medicine, University of Calgary, Calgary, AB T2N 4N1, Canada; 6 Faculty of Kinesiology, University of Calgary, Calgary, AB T2N 1N4, Canada; 7 Cancer Care Alberta, Alberta Health Services, Edmonton, AB T5J 3E4, Canada

**Keywords:** cancer, exercise, eHealth, implementation

## Abstract

A survey was conducted to identify barriers and facilitators to engaging in virtual and in-person cancer-specific exercise during COVID-19. A theory-informed, multi-method, cross-sectional survey was electronically distributed to 192 individuals with cancer investigating preferences towards exercise programming during COVID-19. Respondents had previously participated in an exercise program and comprised two groups: those who had experience with virtual exercise programming (‘Virtual’) and those who had only taken part in in-person exercise (‘In-Person’). Quantitative data were summarized descriptively. Qualitative data were thematically categorized using framework analysis and findings were mapped to an implementation model. The survey completion response rate was 66% (*N* = 127). All respondents identified barriers to attending in-person exercise programming during COVID-19 with concerns over the increased risk of viral exposure. Virtual respondents (*n* = 39) reported: (1) feeling confident in engaging in virtual exercise; and (2) enhanced motivation, accessibility and effectiveness as facilitators to virtual exercise. In-Person respondents (*n* = 88) identified: (1) technology as a barrier to virtual exercise; and (2) low motivation, accessibility and exercise effectiveness as barriers towards virtual exercise. Sixty-six percent (*n* = 58) of In-Person respondents reported that technology support would increase their willingness to exercise virtually. With appropriately targeted support, perceived barriers to accessing virtual exercise—including motivation, accessibility and effectiveness—may become facilitators. The availability of technology support may increase the engagement of individuals with cancer towards virtual exercise programming.

## 1. Introduction

The novel Coronavirus Disease 19 (COVID-19) pandemic significantly increased barriers and disrupted in-person access to healthcare services for immunocompromised populations. Barriers to healthcare delivery from COVID-19 have led to a fundamental shift of patient–clinician interactions from primarily ‘in-person’, to options that include virtual care, telehealth, telemedicine, or ‘eHealth’ [1,2,3]. While eHealth platforms have the potential to provide multidisciplinary care to vulnerable chronic disease populations and overcome remote/rural settings [4], research is still novel and emerging around successful telehealth implementation [5]. 

The disruption to service access negatively impacted individuals with cancer, who are at increased risk for severe complications from COVID-19 due to immunocompromised side effects of cancer therapies, comorbidities and advanced age [6,7]. With the population of individuals diagnosed and living with cancer continuing to rapidly grow worldwide [8,9], there is a widening gap of supportive care services to address the many acute and chronic side effects from cancer and cancer-related treatments [10,11,12]. Supportive care refers to services designed to meet the physical, emotional, social and practical needs of individuals across the cancer spectrum [13]. An extensive body of evidence, including 16 published guidelines from major medical or health-oriented organizations globally, recognize exercise as beneficial for individuals with cancer across the cancer spectrum [14]. Regular exercise results in numerous physiological and psychosocial benefits for cancer survivors, including improved survival outcomes for common cancers, overall quality of life, cancer-related fatigue, cardiorespiratory fitness and muscular strength [14,15,16]. Given the strength of evidence supporting the benefits of exercise for the cancer population, targeted efforts are needed to integrate cancer-specific exercise programming into standard patient care [17,18,19,20], now exacerbated due to increased barriers to exercise presented by COVID-19 [21,22]. 

With the rapid pivot to eHealth virtual platforms, COVID-19 has provided a unique environment to understand cancer survivors’ perspectives on the virtual delivery of exercise programming. Program accessibility is a known barrier identified by individuals with cancer towards engaging in exercise (i.e., transportation, parking, facility type and location, time of day) [23]. While home-based exercise improves accessibility, home programs lack support from exercise professionals and peers, which survivors have identified as significant facilitators towards exercise [24]. There is promise for the use of virtual platforms to deliver accessible cancer-specific exercise programming remotely while maintaining exercise professional and social supports [25,26]. Continuing research during the pandemic has led to initiatives around the large-scale implementation of eHealth platforms focusing on parameters of engagement, such as feasibility, acceptability and efficacy [27,28,29]. Virtual service delivery may provide a means to avoid the unnecessary risks of viral transmission associated with in-person settings [30]; however, the ability of eHealth to meet the exercise needs of people with and recovering from cancer is unclear. Moreover, with the rapid transition to eHealth platforms for cancer supportive care services, there is limited understanding of the best practices for implementing and delivering cancer-specific virtual exercise programming [31].

### 1.1. Research Context of the Clinical Team

#### Alberta Cancer Exercise Hybrid Effectiveness–Implementation (ACE) Study 

The present study was part of the integrated knowledge translation (iKT) series [32] of sub-studies from the Alberta Cancer Exercise Hybrid Effectiveness–Implementation (ACE) study. ACE is an implementation study that proposes a clinic-to-community model of care to support the implementation of cancer-specific, community-based, exercise programming; the protocol and findings are described elsewhere [33,34,35]. The study involves a 12-week exercise program for individuals diagnosed with any cancer. The ACE study pivoted to providing virtual exercise programming during the lockdowns associated with COVID-19.

### 1.2. Objective

This study aimed to understand the facilitators/preferences and barriers towards exercise during COVID-19 to inform ongoing cancer-specific exercise programming. Specific objectives included an understanding of the perspectives of individuals who had previously participated in standardized exercise towards (1) in-person and virtual exercise, and (2) the use of technology to access virtual exercise programming. Findings were intended to inform ACE maintenance programming in Northern Alberta during the pandemic and support future clinical implementation of virtual exercise programming in the cancer setting. 

## 2. Materials and Methods

### 2.1. Study Design

A cross-sectional survey was administered to individuals with cancer who had previously participated in ACE programming at Northern Alberta sites (Edmonton, Grande Prairie, Fort McMurray and Red Deer) either in-person prior to the pandemic, or virtually during the pandemic. A multi-method approach using both quantitative and qualitative data was utilized to provide a more inclusive understanding of the participants’ perspectives towards in-person and virtual exercise during COVID-19, as well as the use of technology to access virtual exercise programming. Survey questions were theory informed and designed based on implementation theory from the Capability, Opportunity, Motivation—Behaviour (COM-B) model [36]. Survey questions were mapped from each of the three model domains/constructs: (1) *capability*—an individual’s psychological (knowledge) and physical capacity (skills) to perform behaviours or activities; (2) *opportunity*—physical (environment) or social factors (interpersonal influences) external to an individual that influence the behaviour; and (3) *motivation*—brain processes that direct behaviour (optimism, habitual and emotional responses, and analytical decision making) [36]. Survey questions included both multiple choice and short answers to comprehensively capture each COM-B model construct. For question mapping and survey questions see Supplementary Material Table S1. Demographic and medical information were previously collected through the ACE study [33]. Ethics approval for this sub-study was granted by the Health Research Board of Alberta: Cancer Committee (HREBA.16-0905) and the intervention component was prospectively registered (NTC02984163). 

### 2.2. Data Collection

Participants were eligible to participate in the survey if: (1) they had enrolled in either fall 2019 or winter 2020 (both in-person), or spring 2020 (virtual) of the ACE 12-week cancer-specific exercise program through sites in Edmonton, Fort McMurray, Red Deer or Grande Prairie; (2) had consented to future contact from the ACE research team; (3) had an active email address; and (4) had completed 12-week and, if applicable, 24-week post program ACE questionnaires. 

‘In-Person’ are respondents who participated in ACE community-based classes prior to COVID-19 (fall 2019, winter 2020). ‘Virtual’ are those who participated virtually (live supervised online classes) during COVID-19 (either ACE spring 2020, or independently). 

Inclusion criteria for the ACE study required participants to: (1) have a diagnosis of cancer of any type; (2) be over the age of 18 years; (3) be able to participate in mild levels of activity at minimum; (4) be pretreatment or receiving active cancer treatment (e.g., surgery, systemic therapy and/or radiation therapy), or have received cancer treatment within the past 3 years or have existing long-term, or have late presenting effects of their cancer treatment (e.g., radiation fibrosis syndrome, lymphoedema, communication deficits related to cancer treatment or incontinence); and (5) be able to provide informed written consent in English. ACE classes involved a combination of aerobic, resistance, balance and flexibility exercises delivered in a standardized circuit-type class setting twice weekly for a minimum of 60 min per session for a 12-week period (approximately 3–4 metabolic equivalent units per session). For intervention description refer to Table 1. The ACE study protocol has been previously reported in detail elsewhere [33].

The ACE study pivoted to providing virtual exercise programming during COVID-19. Virtual exercise programming classes were live, supervised and conducted over zoom within the context of the following parameters: (1) participants were provided with technology support in setting up and using their device in preparation for virtual programming that involved orientation to the virtual platform, evaluating connectivity and troubleshooting any issues related to the virtual environment (i.e., location of device and alignment with the computer camera for facilitating monitoring of exercise performance); (2) all exercise sessions were conducted live over a consistent virtual platform (Zoom); (3) three intensity levels of each exercise (light, moderate, vigorous) were continuously demonstrated for participants by designated exercise professionals; (4) participants were directed to follow appropriate intensity levels and pin the instructor demonstrating the preferred level; (5) exercises were chosen that could be completed in home environments, focused on body weight exercises with consideration of limited space and equipment; (6) each virtual session was monitored by a qualified exercise professional who was responsible for monitoring performance, correcting exercise form and helping troubleshoot any technology issues etc.; (7) exercise resistance bands were provided for participants.

The survey was active from August 2020–September 2020 to coincide with and inform Northern Alberta fall 2020 and winter 2021 ACE exercise programming. The survey was administered electronically through Research Electronic Data Capture (REDCap), a secure, web-based application designed for research study data collection, provided by Women and Children’s Health Research Institute [37], hosted on a secure server in the University of Alberta’s Faculty of Medicine and Dentistry’s data centre. Eligible participants were emailed a secure survey link through REDCap.

### 2.3. Data Analysis

Data from the survey included both continuous and categorical variables. Basic descriptive statistics were generated by REDCap including frequencies, percentages and counts of responses to quantitative questions. Qualitative data from short answer and open-ended questions were analyzed using framework analysis, a form of content analysis to identify patterns in qualitative data, with a defining feature involving matrix outputs of rows and columns of summarized data [38]. Framework analysis provides a practical lens to answer specific questions with actionable outcomes, lending itself well to informing clinical and implementation practices [39]. Three researchers independently coded written responses (KS, MM, ND) into framework tables. After initial coding, researchers collaborated to amend and refine codes, and develop framework tables in relation to patterns of barriers, facilitators and/or preferences towards exercise and technology. Themes were then mapped to respective domains of the COM-B Model to inform implementation strategies for local fall 2020 ACE exercise programming and future clinical practice [40].

## 3. Results

### 3.1. Demographics

A total of 127 cancer survivors responded (66% response rate), with 69% (*n* = 63) aged 55 and older, and 25% (*n* = 32) 40–55 years of age. The average age of respondents was 59 years (SD = 11.4). The most common cancer diagnosis was breast (44%, *n* = 56), followed by digestive cancer (17%, *n* = 22), and head and neck cancer (11%, *n* = 14). The majority of respondents were female (71%, *n* = 90), and 46% (*n* = 58) of all respondents were actively receiving treatment for cancer. Respondents mainly resided in an urban centre (*n* = 93, 73%) or within 15–30 km of an urban centre (*n* = 28, 22%). The average commute to In-Person exercise programming was 14.3 km. Respondent demographics can be viewed in Table 2.

As all survey recipients had previously participated in exercise programming through ACE, we were able to explore the characteristics of non-respondents compared to respondents. Non-respondents were slightly younger with an average age of 57 years of age (SD 11.1) compared to respondents (59 years, SD 11.4). A larger proportion of non-respondents were males (*n* = 29, 45%) compared to respondents (*n* = 37, 29%). Further details on non-respondent demographics can be viewed in Table 2.

### 3.2. Cancer Survivor Exercise Behaviours and Preferences during COVID-19

**Figure 1:**
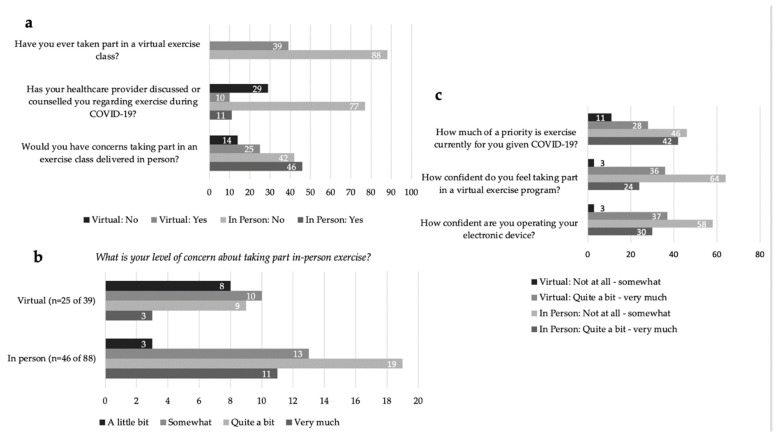
(**a**) Virtual and In-Person exercise preferences and reported exercise counsel by a healthcare professional. (**b**) Virtual and In-Person level of concern regarding in-person exercise in COVID-19. (**c**) Priority of exercise during COVID-19 and confidence ratings accessing virtual exercise and using electronic devices.

In response to the question ‘Would you have concerns about taking part in an exercise class delivered in-person this Fall?’ 56% (*n* = 71 of 127) of all respondents indicated ‘yes’ (Figure 1a). The majority of respondents who identified concern over in-person exercise rated their level of concern for joining in-person exercise programming (fall 2020) from ‘Quite a bit’ to ‘Very Much’ (61%, *n* = 43 of 71) (Figure 1b). All respondents identified barriers to attending in-person exercise programming related to personal safety and concerns over increased risk of COVID-19 exposure and transmission with an in-person exercise setting. The identified risks included: environmental exposure; space and cleaning procedures (e.g., cleaning of equipment, physical distancing, ventilation, sharing of equipment, type of exercise); the burden of masking while exercising; and health-specific risks due to an immunocompromised status from cancer treatments and preexisting comorbidities.

In response to the question, ‘How much of a priority is exercise currently for you given COVID-19?’, responses were mixed with 45% (*n* = 57) reporting ‘Not at all’ or ‘Somewhat’, and 55% (*n* = 70) reporting ‘Quite a bit’ or ‘Very Much’ (Figure 1c). The reported exercise frequency was: 1–2 times per week for 32% of respondents (*n* = 41); 3–4 times per week also for 32% (*n* = 41); greater than 5 times a week for 24% (*n* = 30) and ‘Not at all’ for 12% (*n* = 15). Current exercise environments were identified as: ‘self-exercise alone’ at 71% (*n* = 90); followed by ‘self-directed exercise with others’ (socially distanced walking, running, biking) at 29% (*n* = 37). The three main types of exercise engaged in were reported as: (1) aerobic exercise at 78% (*n* = 99); (2) resistance exercise at 43% (*n* = 54); (3) and flexibility and stretching at 26% (*n* = 33). Thirteen percent (*n* = 17) of respondents were partaking in virtual exercise classes (live or prerecorded). Only 17% (*n* = 21) of respondents reported healthcare provider (HCP)-initiated counseling regarding exercise during COVID-19 (Figure 1a).

We explored the differences in rating barriers and facilitators between those who prioritized exercise (*n* = 70) compared to those who did not prioritize exercise (*n* = 57) during COVID-19. The only notable difference was those who did not prioritize exercise were more confident (self-identified as fairly to completely confident) using their electronic device (*n* = 40 of 57, 71%) compared to those who prioritized exercise (*n* = 36 of 70, 51%).

### 3.3. ACE In-Person Participants and Virtual Exercise Programming

**Figure 2:**
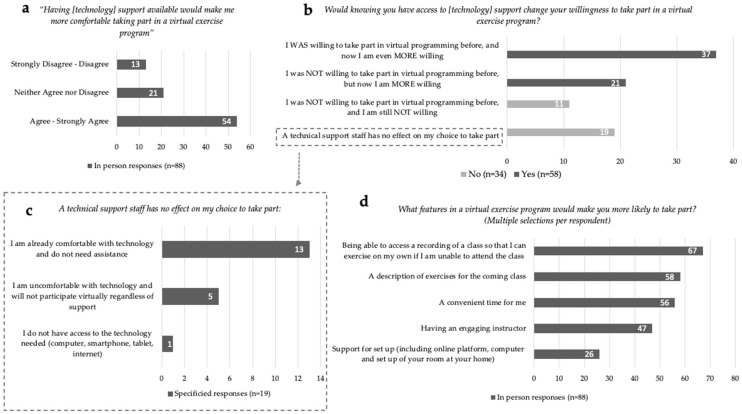
In-Person virtual exercise and technology responses. (**a**) Willingness to take part in an exercise program with technology support available. (**b**) Technology support staff specified responses for unchanged willingness. (**c**) Programming facilitators for virtual exercise engagement. (**d**) Comfort ratings with available technology support staff towards virtual exercise programming.

For In-Person participants (*n* = 88), 73% (*n* = 64) indicated that they were ‘Not at all’ to at most ‘Somewhat’ confident participating in a virtual exercise program (Figure 1c). Communication applications such as Facetime, Skype and Zoom were identified by 20% (*n* = 18) of In-Person respondents to be used at least once a day. The majority, 61% (*n* = 54), ‘Agreed’ or ‘Strongly Agreed’ that the provision of technological support would increase their comfort in taking part in a virtual exercise program (Figure 2a). Responses to the statement, ‘Would knowing you have access to [technology] support change your willingness to take part in a virtual exercise program?’ can be divided into four categories (Figure 2b): (1) 42% (*n* = 37) responded ‘Yes, I WAS willing to take part in virtual programming before, and now I am even MORE willing to take part’; (2) 24% (*n* = 21) indicated ‘Yes, I was NOT willing to take part in virtual programming before, but now I am MORE willing to take part’; (3) 13% (*n* = 11) indicated, ‘No, a technical support staff has no effect on my choice to take part’; (4) 22% (*n* = 19) responded ‘NO—I was NOT willing to take part in virtual programming before, and I am still NOT willing to take part’. Of the respondents who indicated technical support staff had no impact on their participation, 68% (*n* = 13 of 19) stated they were already comfortable with technology and did not need assistance (Figure 2c). Of all In-Person respondents, only 6% (*n* = 5) indicated they would not participate virtually regardless of technology support. Only one respondent indicated they did not have access to the technology needed to participate in exercise virtually. Preferences for virtual exercise program features were identified by In-Person respondents, in order of highest to least priority: (1) access to recordings of classes; (2) exercise descriptions provided prior to the class; (3) convenient class timing; (4) having an engaging instructor; and (5) support for set up (including online platform, computer and set up of exercise space at home) (Figure 2d).

### 3.4. ACE Virtual Participants and Virtual Exercise Programming Experience

ACE spring 2020 online participants who took part in the survey (*n* = 19) responded to the statement ‘I experienced unique benefits taking part in the ACE virtual exercise program during the pandemic’, with 89% (*n* = 17) ‘Agreeing’ or ‘Strongly Agreeing’. These participants were provided with technology support in setting up and using their device to virtually participate in exercise programming, of which 63% (*n* = 12) ‘agreed’ or ‘strongly agreed’ technology support was beneficial. ACE online respondents did not identify any concerns regarding the virtual exercise program itself. Seventy-nine percent (*n* = 15 of 19) of respondents reported they had no difficulties accessing the virtual exercise program. Identified barriers to the virtual exercise programming were reported as a poor internet connection (16%, *n* = 3) and a lack of home exercise equipment (11%, *n* = 2). One respondent reported a lack of comfort using technology and a separate respondent reported their screen was too small to properly follow the virtual program.

### 3.5. Thematic Findings and Implementation Mapping to COM-B Model

For the purposes of exploratory and qualitative analyses, participants were divided into two main groups: (1) respondents with in-person exercise experience alone (‘In-person’, *n* = 88); and (2) respondents with experience exercising in a virtual environment (‘Virtual’, *n* = 39), which included 19 respondents from the spring 2020 session as well as 20 respondents who had participated in-person in the fall 2019 and winter 2020 ACE study sessions as well.

#### 3.5.1. In-Person

**Figure 3:**
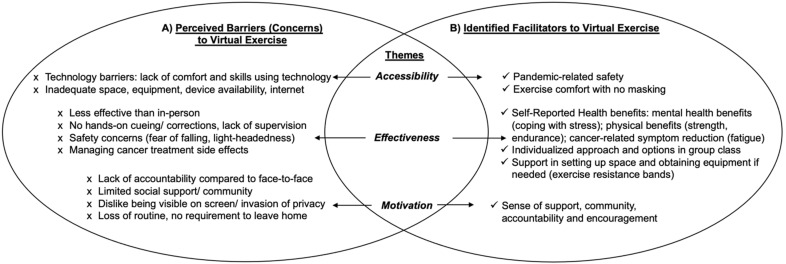
Perceived Barriers and Identified Facilitators towards Virtual Exercise Programming. (**A)**: Perceived Barriers to Virtual Exercise; (**B**): Identified Facilitators to Virtual Exercise.

Individuals with experience with in-person exercise alone identified three main perceived thematic barriers to attending virtual exercise classes: (1) accessibility: lack of technology competency and limited space and exercise equipment at home; (2) effectiveness: virtual exercise programming viewed as less effective than in-person without personalized hands-on cuing, monitoring and corrections from the exercise instructor(s); less effective in managing safety and treatment side effects; and (3) motivation: a perceived lack of accountability with no face-to-face interactions; a lack of social support/community; perceived invasion of privacy (home setting being seen on screen); and a loss of routine. For thematic findings refer to Figure 3A.

**Figure 4:**
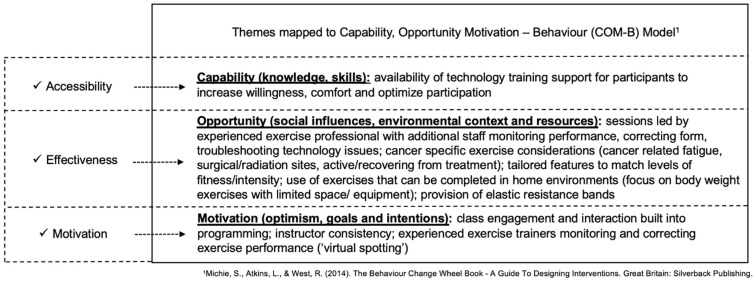
Thematic Findings Mapped to COM-B Model with Clinically Actionable Items to Support Virtual Exercise Implementation [36].

Mapping of themes to the COM-B model corresponded with the following model components: (1) accessibility mapped to Capability: participant identified lack of knowledge and skills towards engaging with technology; (2) effectiveness mapped to Opportunity: a lack of physically present social influences (instructors and other participants) and barriers of the local environmental context and resources; and (3) motivation mapped to Motivation: lack of optimism towards virtual exercise encounters. For thematic mapping to COM-B, refer to Figure 4.

#### 3.5.2. Virtual

Individuals with experience exercising in the virtual environment identified three main thematic benefits to virtual exercise: (1) accessibility: pandemic-related safety; exercise comfort as no masking needed; (2) effectiveness: self-reported physical and mental health benefits including better coping with stress and cancer-related symptom burden reduction; an individualized approach maintained with exercise options in the group class; support for setting up home exercise space and home equipment (resistance bands provided); and (3) motivation: virtual exercise provided sense of community, support and encouragement. For thematic findings refer to Figure 3B.

Mapping of themes to the COM-B corresponded with the following model domains: (1) accessibility mapped to Capability: virtual platform alleviated pandemic-related safety and masking concerns for participants to engage in exercise (2) effectiveness mapped to Opportunity: the virtual class structure facilitated a conducive environment with appropriate resources and social support for participants to engage and exercise safely; and (3) motivation mapped to Motivation: virtual community environment facilitated optimism, and intrinsic goal setting and intentions towards virtual exercise encounters. For thematic mapping to COM-B, refer to Figure 4.

## 4. Discussion

Survey findings showed that a majority of individuals with cancer who had taken part in the ACE program had limited experience engaging with virtual exercise—at a time when they were also uncomfortable attending in-person exercise due to COVID-19. This finding highlights the need for the consideration of alternative modes of exercise programming delivery. Home-based exercise programs have been previously reported to lack community and peer support, leading to reduced adherence and effectiveness in individuals with cancer [41]. Virtual group exercise offers the promise of group support while maintaining social distancing, allowing the convenience of home (no travel time or costs) and increasing accessibility to individuals residing outside of urban centers [29,31]. A study examining the effectiveness of a virtual exercise oncology program, involving 491 cancer participants undergoing antineoplastic therapy between March and June 2020, reported significant benefits for psychological outcomes of improved feelings of support (58.7% increase, *p* < 0.05) and a significant decrease in loneliness (54% decrease, *p* < 0.05) [26].

A primary finding of this survey was that perceived barriers to virtual exercise programming by individuals without virtual exercise experience were identified as facilitators by those who had virtual experience. Virtual programming may be enhanced by considering accessibility and capability options and underlying motivation to facilitate greater engagement. Our survey findings highlight that successful transition from in-person to virtual programming involves more than just offering virtual classes. A recent survey of 593 cancer respondents found strong predictors of cancer survivors’ virtual engagement with HCPs to be access to, and past experiences with, interactive technologies for health-related purposes [42]. Successful transitioning to telehealth for exercise programming was found to be largely influenced by patients’ willingness (motivation) and capability to use technology.

The success of in-person programming for individuals with cancer may not necessarily correlate to successful virtual programs. Implementation efforts may need to specifically address the nuance of virtual versus in-person exercise programming. Specifically, time and resources may need to be allocated for the upskilling of technological competency and confidence, as well as program support (i.e., dedicated staff monitoring virtual exercise participant performance) to preserve service quality in a virtual setting. Exercise professionals may need to adjust their approaches to match the limitations of virtual engagement and allot time to support the setup of an appropriate home virtual exercise environment.

The availability of technology training support for participants could help increase willingness and comfort, and thus optimize motivation. A survey of 377 cancer participants from the Macmillan Move More Northern Ireland (MMNI) exercise program investigated the impact of COVID-19 on the physical activity patterns and attitudes towards digitally supported exercise in individuals with cancer [43]. MMNI pandemic programming offered ‘live’ virtual exercise sessions and a recorded exercise library available on YouTube. Sixty-two percent of respondents (*n* = 233 of 377) reported participating in exercise virtually. Of the 38% of MMNI respondents (*n* = 144) who did not engage with virtual technology, 43% (*n* = 62 of 144) responded they were interested, with participants identifying a lack of technological proficiency/support as a barrier to participation. Given the older age of individuals with cancer at diagnosis [8], it is likely that many individuals have less experience and comfort with virtual environments. Lower computer literacy in combination with age has been reported as a barrier to virtual exercise engagement for individuals with cancer [44]. Thus, an aging cancer population with limited exposure to virtual platforms may warrant additional technology support for effective transition to virtual exercise programming.

A growing body of evidence supports that successful telehealth implementation involves identifying user technology competencies to facilitate participation [45,46]. Providing a standardized technological proficiency assessment tool for initial screening could preemptively identify participants who require further technology support [4]. A recent scoping review examining best practices in the implementation of telehealth-based cancer supportive care included 19 review papers and 23 telehealth guidance documents [28]. Findings concluded that factors related to both the user (cancer population) and the provider (healthcare/supportive care providers) influence the acceptability and effectiveness of telehealth services. The findings suggest that for successful telehealth, providers need to focus on technology competency, device adequacy, participant confidence in utilizing or providing services, and mitigation of the impact on service quality. For clinically actionable items to support virtual exercise implementation see Figure 4.

Strengths of this study included a novel comparison of the perspectives of individuals with cancer towards engaging in in-person and virtual exercise during a pandemic, after previous exercise participation. The online survey format allowed for a greater reach of participants (*n* = 127) and aligned with current COVID-19-related policies for avoiding in-person contact. Consistent with percentages from the overall ACE population, the majority of respondents were female (71%, *n* = 90) and diagnosed with breast cancer (44%, *n* = 56), limiting generalizability to males and other tumor groups. The average age of respondents was 59 years, limiting generalizability to older cancer survivors; however, the average age is similar to the average age of participants (~58 years) in the overall ACE program (*n* = 2270). Non-respondents were slightly younger with a higher proportion of males, with a potential bias in those motivated to respond. All respondents had used an electronic format for patient-reported outcomes during their respective ACE programming (both in person and virtual), so there may be bias in terms of familiarity with the online response format. Additionally, fewer individuals had experience exercising virtually compared to in-person, which offers the potential of skewed responses.

The findings of this survey provide a perspective in understanding how cancer-specific exercise programming delivery can be facilitated to meet the needs of individuals with cancer during a pandemic. The identified differences between In-Person versus Virtual programming highlight the need to create and deliver content matched to both the virtual platforms and to the participants’ levels of capability and confidence in technology. These survey findings indicate the potential benefit of providing dedicated technology support to increase the willingness to participate and engage with novel virtual exercise services.

## Figures and Tables

**Table 1:** Intervention description using the template for description and replication checklist (TIDieR).

Intervention	Alberta Cancer Exercise (ACE) Hybrid Effectiveness Implementation Study [33]
Why	Exercise improves aerobic fitness, muscular strength and cancer-related symptoms
What: Materials	Exercise studio for circuit classes: bands, free weights, mats, bender balls, bosu; Community fitness centre or cancer-specific clinic for group personal training: treadmill, stationary bike, exercise machines (chest press, bicep curl, leg curl/extension, seated row, pullies) free weights and mats
What: Procedures	
Providers	Oversight: exercise physiologist or physical therapist.
	Instructor: qualified exercise professional
How	
	Supervised sessions of either standardized circuit-type class setting or group personal training
Where	Community-based exercise facilities and cancer-specific exercise clinics
Type	Aerobic, resistance, balance and flexibility exercises
Intensity	3–4 metabolic equivalent (MET) units per session (360–480 MET-min/week) Progression of intensity to 4–5 METs over the 12-week duration (480–600 MET-min/week
	2–4 (light to somewhat heavy) on the 11-point Borg Rating of Perceived Exertion Scale
Frequency	Twice weekly
Session time	60 min per session
Overall duration	12-weeks
Tailoring	Adaptations to address cancer-related symptoms, muscular stiffness and dizziness, and prevent adverse events
Trial fidelity	Staff with training and experience in exercise oncology
	Exercise supervision
	Attendance tracked for number of completed sessions
	Monitoring of symptoms (e.g., fatigue, muscle soreness)
	Recording of minor and serious adverse events

**Table 2:** Baseline Demographic and Medical Data.

Respondent Characteristics	In-Person Exercise (Spring 2019–Winter 2020)	Virtual Exercise (Spring 2020)	Total Respondents	Non- Respondents
	*n* = 88, No. (%)	*n* = 39, No. (%)	*n* = 127, No. (%)	*n* = 65, No. (%)
	Sex			
Male	29 (33.0)	8 (20.5)	37 (29.1)	29 (44.6)
Female	59 (67.0)	31 (79.5)	90 (70.9)	36 (55.4)
	Age			
26–39	7 (7.8)	1 (2.6)	8 (6.3)	5 (7.7)
40–54	18 (20.5)	14 (35.9)	32 (25.2)	22 (33.8)
55–69	47 (53.4)	16 (41.0)	63 (49.6)	30 (46.2)
>70	16 (18.2)	8 (20.5)	24 (18.9)	8 (12.3)
Average Age (Years, Standard deviation)	58.7 (11.5)	59.0 (11.3)	59.0 (11.4)	56.7 (11.1)
	Tumor Type			
Blood	12 (11.1)	1 (5.3)	13 (10.2)	3 (4.6)
Breast	35 (39.8)	21 (53.8)	56 (44.1)	17 (26.2)
Gastrointestinal	16 (18.2)	6 (15.4)	22 (17.3)	7 (10.8)
Genitourinary	3 (3.4)	2 (5.1)	5 (3.9)	1 (1.5)
Gynecological	2 (2.3)	2 (5.1)	4 (3.1)	5 (7.7)
Head and neck	11 (12.5)	3 (7.7)	14 (11.0)	7 (10.8)
Lung	1 (1.1)	1 (2.6)	2 (1.6)	2 (3.1)
Neurological	6 (6.8)	1 (2.6)	7 (5.5)	6 (9.2)
Skin	1 (0.9)	0 (0.0)	1 (0.8)	3 (4.6)
Other	1 (0.9)	2 (10.5)	3 (2.4)	2 (3.1)
	Currently receiving treatment (while in exercise program)			
Yes	39 (44.3)	19 (48.7)	58 (45.7)	31 (47.7)
No	49 (55.7)	20 (51.3)	69 (54.3)	34 (52.3)
	Cancer Treatment (received while in exercise program)			
Chemotherapy	17 (19.3)	7 (17.9)	24 (18.9)	10 (15.4)
Radiation	7 (6.5)	0 (0.0)	7 (5.5)	4 (6.2)
Hormone Therapy	10 (11.3)	11 (28.2)	21 (16.5)	10 (15.4)
Biological Therapy	0 (0.0)	1 (2.6)	1 (0.8)	4 (6.2)
Other	12 (13.6)	2 (5.1)	14 (11.0)	6 (9.2)
	Cancer Treatment (completed)			
Chemotherapy	57 (64.8)	18 (46.1)	75 (59.1)	40 (61.5)
Radiation	47 (53.4)	25 (64.1)	72 (56.7)	34 (52.3)
Hormone Therapy	6 (6.8)	2 (5.1)	8 (6.3)	3 (4.6)
Biological Therapy	0 (0.0)	0 (0.0)	0 (0.0)	2 (3.1)
Surgery	58 (65.9)	30 (76.9)	88 (69.3)	47 (72.3)
Other	12 (13.6)	2 (5.1)	14 (11.0)	6 (9.2)
	Location of Residence			
Edmonton (urban)	62 (70.5)	31 (79.5)	93 (73.2)	53 (81.5)
Catchment area 15–30 km	24 (27.3)	4 (10.3)	28 (22.0)	6 (9.2)
Catchment area 30–100 km	2 (2.3)	3 (7.7)	5 (3.9)	5 (7.7)
Rural > 100 km	0 (0.0)	1 (2.6)	1 (0.8)	1 (1.5)
Average km Commute	14.3 km	N/A	N/A	19.0 km

## Data Availability

The data on repository will be available on request to the corresponding author.

## Supplementary Materials

The following supporting information can be downloaded at: https://www.mdpi.com/article/10.3390/curroncol29100529/s1, Table: S1. Capability, Opportunity, Motivation-Behaviour (COM-B) Model1 Survey Question Mapping.
