# Supplementary material for: Virtual or In-Person: A Mixed Methods Survey to Determine Exercise Programming Preferences during COVID-19
Source: Curr Oncol. 2022 Sep 20;29(10):6735–48. doi: 10.3390/curroncol29100529 (PMC9601145; doi:10.3390/curroncol29100529)
Supplement: Supplementary file 1 [file curroncol-29-00529-s001.zip › curroncol-1858846-supplementary.pdf]

<table>
<tr><td colspan="3">Table S1. Capability, Opportunity, Motivation-Behaviour (COM-B) Model [36] Survey Question Mapping</td></tr>
<tr><th>COM-B [36] Components and domains</th><th colspan="2">Responses by Group: In-Person* and Virtual**</th></tr>
<tr><td rowspan="4">Component:
<u>Capability</u>
Domains:
Knowledge
Skills (Physical, Psychological)
Memory, Attention and Decision Processes</td><th>In-Person and Virtual</th><th>In-Person Only</th></tr>
<tr><td rowspan="3">• How are you currently exercising?
• What type(s) of exercise are you currently taking part in?
• How confident are you in operating your electronic device?
• How often do you use programs such as FaceTime, Skype, and Zoom to connect with others?
• What do you use for your virtual communications?</td><td>• How confident do you feel about taking part in a virtual exercise program?
• What device(s) would you use if you took part in a virtual program?</td></tr>
<tr><th>Virtual Only</th></tr>
<tr><td>• How confident do you feel about taking part in a virtual program again?</td></tr>
<tr><td rowspan="4">Component:
<u>Opportunity:</u>
Domains:
Social Influences
Environmental Context and Resources</td><th>In-Person and Virtual</th><th>In-Person Only</th></tr>
<tr><td>• Have you ever taken part in a virtual exercise class?
• Has your healthcare provider discussed or counselled you regarding exercise during COVID-19?
• What exercise equipment do you have access to?
• Would you have concerns about taking part in an exercise class delivered in-person this fall?
• What is your level of concern about taking part in in-person exercise?
• What are your concerns about in-person exercise classes?
• Would you have concerns about taking part in a virtual exercise class this fall?
• What is your level of concern about taking part in virtual exercise?
• What are your concerns about virtual exercise classes?
• What type(s) of exercise programming would you be open to taking part in this fall?
• If you were asked to complete fitness testing, how would you prefer to complete these tests?
• Have you ever taken part in a virtual exercise class?
• Would your family or friends be supportive of you engaging in virtual/in-person exercise programming?</td><td>• ACE [33] staff can provide support to help you get familiar with using the virtual programming, and to ensure you are set up at home. Please indicate your agreement with the following statement: "Having support available would make me more comfortable taking part in a virtual exercise program."
• Would knowing you have access to support change your willingness to take part in a virtual exercise program?
• Having support staff to assist you would not change your choice to take part in virtual exercise because…
• Would someone be available to assist you with technology if you were participating in a virtual exercise program?
• What features in a virtual exercise program would make you more likely to take part? Please select all that apply. Please specify what other feature(s) would make you more likely to participate.
• What difficulties would you anticipate if you were to take part in a virtual exercise program?
• Would you have another person at home during the virtual exercise classes who could assist you if required (with non-technology issues)?</td></tr>
<tr><th colspan="2">Virtual Only</th></tr>
<tr><td colspan="2">• If you were to participate in a virtual exercise program again, would you use the same device to connect to the class?
• What different device(s) would you use? Please select all that apply.
• Did you find having support available in setting up and using your device to participate in the class beneficial?
• Please rate the following statement: “I experienced unique benefits taking part in the ACE Spring virtual exercise program during the COVID-19 pandemic.” Please let us know what the unique benefits were that you experienced.
• Did you experience any of the following difficulties during the virtual exercise program? Please select all that apply.
• Are there any features of the ACE virtual exercise program that you would change?</td></tr>
<tr><td rowspan="2">Component:
<u>Motivation:</u>
Domains:
Intentions
Goals
Beliefs about capabilities
Optimism</td><th colspan="2">In-Person and Virtual</th></tr>
<tr><td colspan="2">• How much of a priority is exercise currently for you given COVID-19?
• On average, how many times per week did you exercise in the last month? (Please only count days where you exercised for at least 10 minutes at a moderate intensity level)
• On average, how many minutes per week did you exercise (at a moderate or vigorous intensity) in the last month?
• To meet Alberta Health Services guidelines and accommodate all participants, a likely program option for the fall will be having one class a week of virtual exercise, and the other as in-person exercise OR only virtual exercise classes. How willing would you be to participate in a combined exercise program where you attended one day in-person and the second day virtually?</td></tr>
<tr><td colspan="3">* In-Person: previous in-person exercise experience alone (n=88)
** Virtual: experience exercising in a virtual environment (n=39)</td></tr>
</table>
